# Combined treatment of marizomib and cisplatin modulates cervical cancer growth and invasion and enhances antitumor potential *in vitro* and *in vivo*

**DOI:** 10.3389/fonc.2022.974573

**Published:** 2022-08-30

**Authors:** Ziruizhuo Zhang, Songcheng Zhang, Bingjie Lin, Qixin Wang, Xiaojing Nie, Yonghua Shi

**Affiliations:** ^1^ Department of Pathology, School of Basic Medical Sciences, Xinjiang Medical University, Urumqi, Xinjiang, China; ^2^ Department of Pediatrics, Nanyang Chinese Medicine Hospital, Nanyang, Henan, China; ^3^ Xinjiang Key Laboratory of Molecular Biology for Endemic Diseases, Xinjiang Medical University, Urumqi, Xinjiang, China

**Keywords:** proteasome inhibitor, marizomib, cisplatin, drug resistance, cervical cancer

## Abstract

Proteasome inhibition is an attractive approach for anticancer therapy. Cisplatin (cis-diamminedichloroplatinum, CDDP) is widely used as a standard chemotherapy drug in the treatment of solid malignant tumors, such as cervical cancer, ovarian cancer, colorectal cancer, and lung cancer. However, the development of CDDP resistance largely limits its clinical application. Proteasome inhibitors may enhance traditional chemotherapy agent-induced cytotoxicity and apoptosis. Marizomib (NPI-0052, salinosporamide A, Mzb), a second-generation proteasome inhibitor, shows synergistic anticancer activity with some drugs. Currently, the effect of Mzb on cervical cancer cell proliferation remains unclear. In this study, we explored the role of Mzb in three cervical cancer cell lines, HeLa, CaSki, and C33A, representing major molecular subtypes of cervical cancer and xenografts. We found that Mzb alone showed noteworthy cytotoxic effects, and its combination with CDDP resulted in more obvious cytotoxicity and apoptosis in cervical cancer cell lines and xenografts. In order to investigate the mechanism of this effect, we probed whether Mzb alone or in combination with CDDP had a better antitumor response by enhancing CDDP-induced angiopoietin 1 (Ang-1) expression and inhibiting the expression of TEK receptor tyrosine kinase (Tie-2) in the Ang-1/Tie-2 pathway, FMS-like tyrosine kinase 3 ligand (Flt-3L) and stem cell factor (SCF) as identified by a cytokine antibody chip test. The results suggest that Mzb has better antitumor effects on cervical cancer cells and can sensitize cervical cancer cells to CDDP treatment both *in vitro* and *in vivo.* Accordingly, we conclude that the combination of CDDP with Mzb produces synergistic anticancer activity and that Mzb may be a potential effective drug in combination therapy for cervical cancer patients.

## Introduction

The survival of tumor cells depends on proteasome activity. Therefore, proteasome degradation plays a key role in the survival of cancer cells (1, 2). Proteasome inhibitors (PIs) have been shown to exert significant anticancer activity in a variety of cancer treatments (3). Marizomib (NPI-0052, salinosporamide A, Mzb), an orally irreversible second-generation PI, was originally isolated as a natural product from sedimentary marine actinomycetes (4, 5). Structurally, Mzb contains a unique γ-lactam ring and β-lactone ring (6). Pharmacologically, Mzb can selectively and efficiently inhibit the 20S proteasome and pass through the blood−brain barrier and has been approved for clinical trials (7, 8). Mzb inhibits mitochondrial complex II-dependent respiration in triple-negative breast cancer (TNBC) cells, inhibits mitochondrial ATP production, and inhibits the epithelial-mesenchymal transition (EMT) to reduce tumor metastasis *in vivo.* Furthermore, Mzb activates multiple caspases (caspase 3, caspase 8 and caspase 9), scavenges reactive oxygen species (ROS), and finally induces apoptosis (9, 10). However, the specific anticancer molecular mechanism of Mzb is still not clear, and to our knowledge its anticancer effect on cervical cancer has not yet been reported.

Cisplatin (cis-diamminedichloroplatinum, CDDP) is a platinum drug. It is widely used as a standard chemotherapy drug in the treatment of solid malignant tumors such as cervical cancer, ovarian cancer, colorectal cancer, and lung cancer (11). CDDP usually binds to genomic DNA (gDNA) or mitochondrial DNA (mtDNA), causing DNA damage, preventing DNA, mRNA or protein production, interfering with DNA repair, inhibiting DNA replication and transcription, activating multiple signal transduction pathways, and ultimately causing cell cycle arrest and apoptosis (12, 13). Apoptosis is the main cellular response to chemotherapy drugs, including CDDP. Apoptosis results from the activation of the caspase family of aspartate-specific proteases, in which caspase 3 plays an important role in CDDP-induced apoptosis. Additionally, PARP (poly (ADP ribose) polymerase) mediated cell death have been identified as novel targets for cancer therapy (14). Although CDDP damages cancer cells by a variety of mechanisms, the accumulation of CDDP in cells decreases with time. Cancer cells develop high resistance to CDDP due to increased activation or modification of the DNA damage repair system, changed pathway of apoptosis, activation of the epithelial mesenchymal transformation and DNA methylation (15–17). In addition, CDDP has some limitations in terms of multiorgan toxicity, and cell resistance cannot be overcome simply by increasing the dose (18). Overall, CDDP resistance largely limits its clinical application. Recently, CDDP in combination with other compounds or drugs has been reported to be more effective than CDDP alone (19–21). Therefore, testing the combination of Mzb and CDDP in some solid cancers *in vitro* and *in vivo* is a promising approach for therapeutic development.

Cervical cancer is one of the most common malignant tumors in women, following breast cancer, colorectal cancer and lung cancer, and its morbidity and mortality rank fourth among gynecological malignancies (22). CDDP-based combination chemotherapy is the standard chemotherapy regimen for locally advanced and metastatic cervical cancer (23, 24). The 5-year-old overall survival (OS) rates for early-stage, locally advanced, and metastatic cervical cancer are approximately 92%, 65%, and 17%, respectively (25). Although early-stage cervical cancer is usually curable, there is still no satisfactory treatment for locally advanced, recurrent or metastatic cases. Therefore, it has become critical to develop a new anticancer strategy to overcome CDDP resistance and reduce its toxic side effects.

In this preclinical study, we investigated the effect of Mzb combined with CDDP on cervical cancer cell proliferation and apoptosis *in vitro* and *in vivo* and found that the combination of the two drugs had a synergistic effect on the treatment of cervical cancer. Specifically, we demonstrate that Mzb sensitizes cervical cancer cells to the cytotoxic effect of CDDP by upregulating Ang-1 and downregulating the expression of SCF, Flt-3L and Tie-2.

## Materials and methods

### Cell lines and cell culture

Three molecular subtypes of human cervical cancer cell lines were used in this study, as well as a human cervical epithelial immortalized cell line, H8. The CaSki cell line was purchased from the Institute of Basic Medical Sciences, Chinese Academy of Medical Sciences, and the C33A cell line was purchased from Procell Life Science & Technology Co., Ltd. HeLa and H8 cells were cryopreserved in the National Pathology Laboratory of Xinjiang Medical University. HeLa, C33A and H8 cells were routinely cultured in Dulbecco’s modified Eagle’s medium (DMEM, HyClone, USA), and CaSki cells were maintained in roswell park memorial institute (RPMI)-1640 medium (HyClone, USA), which were supplemented with 10% fetal bovine serum (FBS, Gibco, USA), 100 units/mL penicillin, and 100 mg/mL streptomycin (Gibco, USA). Cells were cultured at 37 °C in a humidified atmosphere of 5% CO_2_.

### Antibodies and reagents

The antigens were detected using rabbit polyclonal or monoclonal primary antibodies against PARP (Cell Signaling Technology, Cat #9532, RRID: AB_659884), caspase 3 (Cell Signaling Technology, Cat #9662, RRID: AB_331439), Ang-1 (Abcam, Cat #ab183701, RRID: AB_2920795), Flt-3L (Abcam, Cat #ab52648, RRID: AB_2104974), Tie-2 (Abcam, Cat #ab221154, RRID: AB_2920796), SCF (Abcam, Cat #ab52603, RRID: AB_870641), GAPDH (Proteintech, Cat #10494-1-AP, RRID: AB_2263076), Ki-67 (ZSGB-Bio, Cat #ZM-0166, RRID: AB_2890067), and the alkaline phosphatase-conjugated affinipure goat anti-rabbit IgG (Proteintech, Cat #SA00002-2, RRID: AB_2752246). CDDP (P4394) was obtained from Sigma−Aldrich LLC. Mzb was purchased from Cayman Chemical (10007311) and MedChemExpress (MCE, HY-10985). PE Annexin V Apoptosis Detection Kit I was purchased from BD Biosciences (Cat 559763). An immunohistochemical universal type two-step method detection kit (PV-9000) and diaminobenzidine (DAB) coloration (ZLI-9018) were purchased from Zhongshan Golden Bridge (Beijing, China).

### Drug preparation

CDDP lyophilized powder was dissolved with phosphate buffered saline (PBS) to make 11.11 mmol/L solution, and Mzb lyophilized powder was dissolved with dimethyl sulfoxide (DMSO, neoFroxx GmbH, Germany) to make 106.23 μmol/L solution. The solutions were stored at -20°C, and diluted with PBS to the desired concentration when used.

### Cytotoxicity assay

Cell cytotoxicity assays were performed using a Cell Counting Kit-8 (CCK-8, Bioss Inc., China) following the manufacturer’s instructions. Cells in the logarithmic growth phase were used for experiments. Cells were seeded in 96-well plates at a density of 5 × 10^3^ per well (200 μL per well) and cultured for 24 h at 37°C. Cells were either allowed to grow in medium alone or in medium containing increasing concentrations of Mzb, CDDP, or a combination of the two drugs, and each group was prepared with 5 replicate wells. Seventy-two hours later, the cells were observed and photographed by optical microscopy. Then, 20 μL of CCK-8 working solution was added to each well, and the cells were incubated at 37°C and 5% CO_2_ for 2 hours. The absorbance of each well was measured at 450 nm with a microplate reader and the cell viability curve was plotted.

### Determination of synergy

Cells were seeded (5 × 10^3^/well) in different 96-well plates and allowed to recover overnight. The cells were treated for 72 h with varying doses of CDDP alone or with varying doses of a nonconstant proportion of the two drugs together. Nontreatment was used as a control. Following the completion of CCK-8 assays, the data were analyzed, and combination indices (CIs) were determined based on the method of Chou and Talalay (26) using CompuSyn V1.0 software (ComboSyn, Inc.). CI values below 1.0 are considered evidence of synergy.

### Colony formation assay

Cells were seeded in 12-well plates at 2 × 10^3^ cells per well. After 24 h of incubation, the cells were incubated with Mzb at 0, 0.01 μM or 0.025 μM for 72 h. Then, the cells were cultured in drug-free culture medium for 2 weeks until a clone was visible with the naked eye. After that, the cells were fixed and stained with methanol and crystal violet for 10 min and photographed. The number of clones greater than 10 cells was counted using a low-power microscope. Clonal formation rate = (number of clones/number of seeded cells) × 100%. Each experiment was performed in triplicate.

### Western blotting

After the treatment, the cells were washed twice with ice-cold PBS. Cells were scraped and aliquoted into 1.5 mL centrifuge tubes on ice and centrifuged at 1000 rpm for 5 min. Afterward, the supernatant was discarded, lysis buffer was added, and the cell pellet was lysed in lysis buffer for 30 min at 4°C. The solution was centrifuged at 12000 rpm for 15 min, and the supernatant was collected as the cell lysate. For cervical cancer xenograft tissue, the tissues of each group were washed with ice-cold PBS after dissection to remove extra blood. The samples were then chopped into grinding tubes, lysis buffer was added, and the samples were ground with a homogenizer for 30 min, and centrifuged at 12000 rpm for 20 min at 4°C. The lysates were electrophoresed on 10% or 12% SDS−PAGE and transferred to polyvinylidene fluoride (PVDF, Sigma−Aldrich LLC.; 3010040001) membranes, followed by western blotting with the primary antibodies overnight at 4°C (PARP, 1:1000, capsase 3, 1:1000, GAPDH, 1:10000, Ang-1, 1:10000, Tie-2, 1:1000, Flt-3L, 1:100000, SCF, 1:10000). After washing with TBST buffer, the membrane was incubated with the alkaline phosphatase-conjugated secondary antibody against rabbit IgG (1:1000) for 2 hours at room temperature. Finally, immunoreactivity was detected with alkaline phosphatase (AP) chromogenic substrate (Thermo Fisher Scientific Inc.; WP20001) reagent.

### Flow cytometry and Phycoerythrin (PE) annexin V staining assay

The experiment was performed as the following. Cervical cancer cell lines were seeded in 6-well plates and treated with Mzb (0.025 μM), CDDP (80 μM) or the combination of the two drugs for 16 h or 24 h. Cells were trypsinized, resuspended in the medium with 10% FBS, and centrifuged at 1500 rpm for 5 min at 4 °C. Cells were then washed with PBS for two times, resuspended in 300 μL buffer with 5 μL of Phycoerythrin (PE) Annexin V and 5 μL of 7-Amino-Actinomycin D (7-AAD) staining solution, and then transferred into new 5 mL culture tubes with filter. The tubes were gently vortexed and incubated for 15 min at RT (25 °C) in the dark, then the samples were analyzed by flow cytometry within 1 h. Early apoptotic cells with intact membranes are subject to PE Annexin V staining. Viable cells with intact membranes resist 7-AAD, only the membranes of dead cells are subject to 7-AAD. Unstained cells were used as a negative control and untreated cells were used as a control for treated cells. Then test was performed on a BD FACS Aria flow cytometer (BD Biosciences) and analysis of data was performed using the FlowJo V10.8.1.

### Cytokine antibody chip

The HeLa cell line that was relatively insensitive to CDDP was identified and treated with CDDP alone (40 μM) or in combination with Mzb (0.01 μM) for 48 h based on the IC50 value of Mzb monotherapy in HeLa cells. The untreated and CDDP groups were used as the control and positive control groups. A relatively quantitative human cytokine antibody chip (Guangzhou Raybiotech Life, Inc., GSH-CAA-440) was used to measure 440 cytokines, and bioinformatics analysis was performed using protein−protein interaction (PPI) networks in the STRING database (string-db.org, V11.5, minimum required interaction score: default value 0.4).

### Xenografts and tumor growth analysis *in vivo*

For the human HeLa cell line xenograft model, 4 × 10^6^ cells were prepared in 200 μL PBS and injected into the right axillary subcutaneous tissue of 3 to 5-week-old immunocompromised BALB/C female nude mice. Once the tumor size reached approximately 60 mm^3^, the mice were randomly divided into 6 groups (4/group) and then treated with PBS (once per week, by i.p. injection), CDDP (6 mg/kg, once per week, by i.p. injection), Mzb (0.075 mg or 0.15 mg/kg, twice per week, by i.p. injection), and a combination of two drugs (CDDP 3 mg/kg, Mzb 0.075 mg/kg; CDDP 6 mg/kg, Mzb 0.075 mg/kg; twice per week, by i.p. injection) for 2 weeks. Tumor growth was measured three times per week using a digital caliper. After 14 days, the mice were euthanized by cervical dislocation, and the tumors were dissected with surgical scissors. To calculate the tumor volume, the following formula was used: tumor volume = [L × W ^2^]/2, where W = tumor width and L = tumor length (3). The animal experiment setup was provided by the Animal Laboratory Center of Xinjiang Medical University.

### Hematoxylin-eosin staining of pathological sections

The dissected cervical tumors were cut into 0.4 cm x 0.4 cm x 0.4 cm tissue pieces and fixed in 10% formalin fixative for approximately 1 week. After dehydration in a 95% ethanol solution, the tissue samples were clarified in xylene, embedded in paraffin, and cut into 4 μm paraffin sections with an automatic microtome. The sections were stained with hematoxylin and eosin (H&E). and sealed with neutral resin. The changes in tumors among the groups, such as hemorrhage and necrosis, were observed under a microscope.

### Immunohistochemistry

We prepared 4 μm thick sections for each group of tumor tissues. The sections were labeled using anti-Ang-1, anti-Tie-2, anti-SCF, and anti-Flt-3L primary antibodies (Abcam; at 1:200, 1:50, 1:200, 1:180, respectively) at 4°C overnight and then incubated with a secondary antibody (goat anti-rabbit IgG; Zhongshan Golden Bridge, Beijing, China) for 1 h at room temperature. Following DAB coloration, hematoxylin counterstaining, dehydration and clearing in xylene, the slides were mounted. Staining intensity and positive area were analyzed by the average density (Average Optical Density, AOD) using image J software (V1.8.0.172). AOD =Integral Optical Density (IOD)/Positive area.

### Statistical analysis

Statistical analysis was performed using GraphPad Prism 7 (GraphPad Software, CA, USA). All values are presented as the mean ± standard deviation (SD). P values < 0.05 were considered to be statistically significant. Student’s t test (two-tailed) or ANOVA (Dunnett’s multiple comparison post-test) were used to analyze the difference between the drug treatment groups and the control group.

## Results

### Mzb inhibits the proliferation of cervical cancer cells *in vitro*

**Figure 1:**
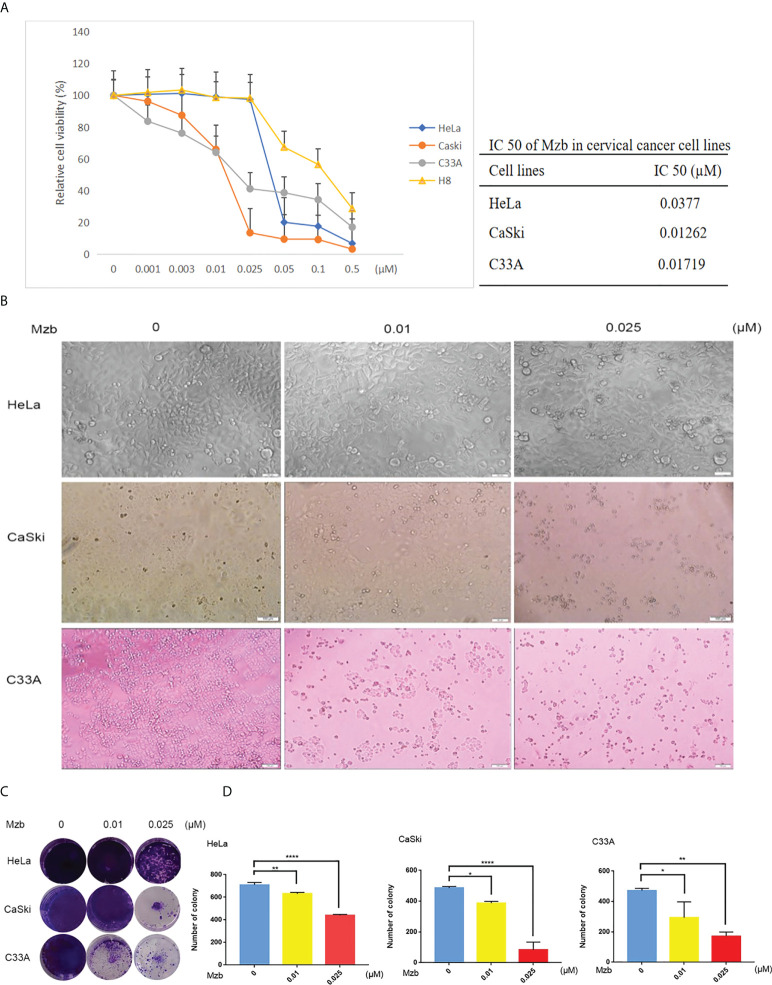
Marizomib shows cytotoxic effects on cervical cancer cells. **(A)** Cytotoxic effects of marizomib on cervical cancer cells in CCK-8 assays. Three human cervical cancer cell lines, HeLa, CaSki, and C33A, and a cervical epithelial immortalized cell line, H8, were treated with 0, 0.001 μM, 0.003 μM, 0.01 μM, 0.025 μM, 0.05 μM, 0.1 μM or 0.5 μM marizomib for 72 h and then used for CCK-8 assays. The absorbance of each well was measured at 450 nm and the cell viability curve was plotted. The IC50 values of marizomib in cervical cancer cell lines were listed. **(B)** Marizomib could cause morphological changes and decreased cell proliferation in cervical cancer cells. **(C)** The three cervical cancer cell lines HeLa, CaSki and C33A were incubated with 0, 0.01 μM or 0.025 μM marizomib for 72 h in a 12-well plate. The cells were then incubated for two weeks with drug-free medium, stained with crystal violet and imaged. **(D)** Count and plot cell clones of **(C)**. The data were presented as the mean ± SD. *P<0.05, **P ≤ 0.01, ****P ≤ 0.0001, by ANOVA (Dunnett’s multiple comparison post-test).

To assess the antitumor effects of Mzb on cervical cancer cells, three cervical cancer cell lines, HeLa, CaSki, and C33A, were used, which together represent the major molecular subtypes of cervical cancer (**Table 1**), and the immortalized cervical epithelial cell line H8 was used as a control. These cells were treated with Mzb at the indicated concentrations of 0.001 μM to 0.5 μM for 72 h and then examined using a CCK-8 assay. The results showed that Mzb reduced the viability of cervical cancer cells in a dose-dependent manner. Meanwhile, cervical cancer cells were more sensitive to Mzb than cervical epithelial immortalized cells (**Figure 1A**). The IC50s of Mzb ranging from 12.62 nM (CaSki) to 37.7 nM (HeLa) were much lower than that of H8 (66.91 nM). Morphological changes in cells after treatment for 72 hours confirmed the cytotoxic effect of Mzb (**Figure 1B**). Since the IC50 values were around the dose of 0.01 μM and 0.025 μM, within all cell lines, we only showed the data for these two doses.

**Table 1:** Molecular classification of human cervical cancer and epithelial immortalized cell lines.

Cell line	Tumor type	HPV16	HPV18	Transfer
HeLa	AC	−	+	−
CaSki	SC	+	+	IM
C33A	SC	−	−	−
H8	−	−	−	−

AC, adenocarcinoma; SC, squamous carcinoma; IM, intestinal metastasis.

To further investigate the inhibitory effect of Mzb on cervical cancer cell proliferation, a cellular colony formation test was performed. Cervical cancer cells were treated with Mzb at concentrations of 0, 0.01 μM, and 0.025 μM for 72 h and then cultured in drug-free medium for about two weeks. The numbers of clones in the Mzb treatment group were dose-dependently decreased compared to those in the control group, which showed that Mzb significantly inhibited the cellular proliferation (**Figures 1C, D**). These data show that Mzb has a strong inhibitory effect on the proliferation of cervical cancer cells, regardless of molecular subtype.

### Mzb induces apoptosis of cervical cancer cells

**Figure 2:**
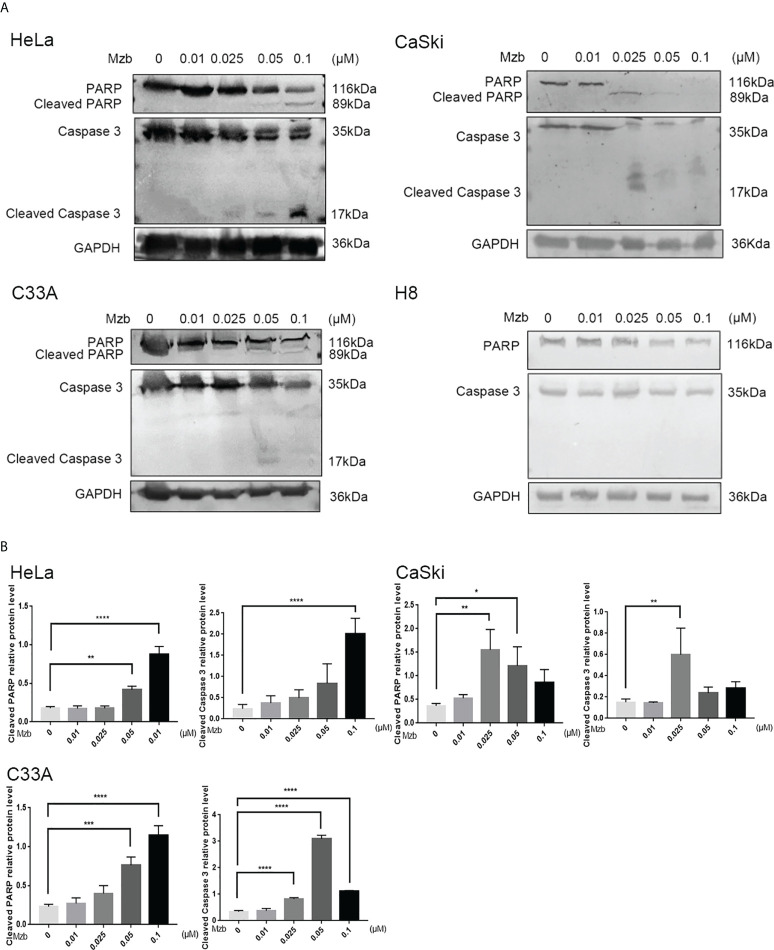
Marizomib induces apoptosis in cervical cancer cells. **(A, B)** The cervical cancer cell lines HeLa, CaSki, and C33A and cervical epithelial immortalized H8 cells were treated with marizomib (0, 0.01 μM, 0.025 μM, 0.05 μM, or 0.1 μM) for 16 h or 24 h. Then, whole-cell lysates were subjected to SDS−PAGE and immunoblotted with antibodies against PARP and caspase 3 to detect apoptosis. GAPDH was used as the loading control. *P<0.05, **P ≤ 0.01, ***P ≤ 0.001, ****P ≤ 0.0001, by ANOVA (Dunnett’s multiple comparison post-test).

Mzb has been reported to exert significant antitumor activity as a single agent or as part of a combination of therapies in various clinical trials, including glioblastoma therapy (27). To detect whether Mzb can induce apoptosis in human cervical cancer cells, HeLa and CaSki cells were treated for 24 h with Mzb at concentrations of 0, 0.01 μM, 0.025 μM, 0.05 μM and 0.1 μM, and the cells were collected to extract proteins for western blotting. H8 was used as a control. C33A cells were collected for protein extraction after Mzb treatment for 16 h. The results showed that Mzb induced cleaved PARP and caspase 3 in the cervical cancer cell lines in a dose-dependent manner, but not in cervical epithelial immortalized cells (**Figures 2A, B**). The results showed that Mzb could induce apoptosis in the cervical cancer cell lines in a dose-dependent manner but had no obvious proapoptotic effect on immortalized cervical epithelial cells. Taken together, these results suggest that Mzb alone could trigger apoptosis in cervical cancer cells.

### Mzb enhances the cytotoxic effect of CDDP on cervical cancer cells

**Figure 3:**
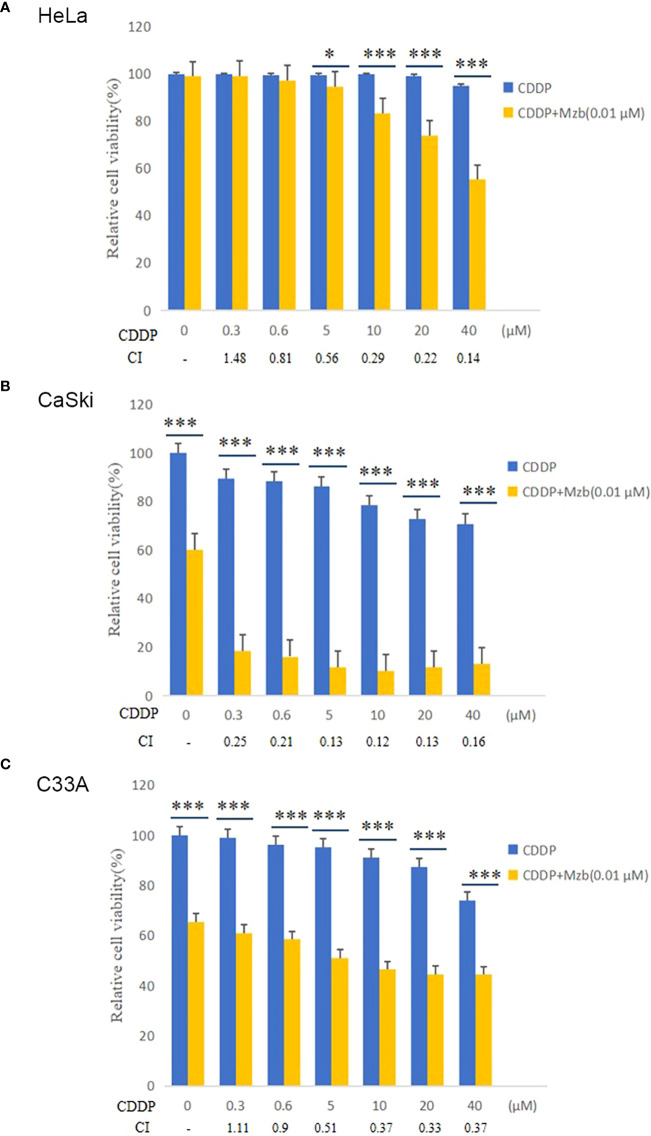
Marizomib enhances the cytotoxic effect of CDDP on cervical cancer cells. **(A–C)** The cervical cancer cell lines HeLa, CaSki and C33A were treated with CDDP at the indicated concentrations with or without 0.01 μM marizomib for 72 h. Cell viability was then measured by CCK-8 assays. CI values were determined using CompuSyn software. The data were presented as the mean ± SD. *P<0.05, ***P ≤ 0.001, by t test.

To investigate whether Mzb and CDDP have synergistic effects on cervical cancer cells, the cells were cultured in increasing concentrations of 0, 0.3 μM, 0.6 μM, 5 μM, 10 μM, 20 μM or 40 μM CDDP alone or in combination with 0.01 μM Mzb for 72 h, and cell proliferation was assessed using a CCK-8 assay. The results showed that the cell viabilities in the combination group were significantly reduced compared to CDDP alone (**Figures 3A–C**). The combination indices (CIs) for most combinations were far lower than 1.0, indicating synergistic effects on cervical cancer cells (**Figures 3A–C**; **Supplementary Tables S1–S3**). In addition, the synergistic cytotoxic effects of the tested cervical cancer cell lines treated with the combination of Mzb and CDDP showed some divergence. This implies that the sensitivity of Mzb is different for different subtypes of cervical cancer cell lines. These results demonstrate that Mzb could sensitize the cytotoxicity of CDDP on the tested cervical cancer cell lines.

### Mzb sensitizes cervical cancer cells to CDDP-induced apoptosis

**Figure 4:**
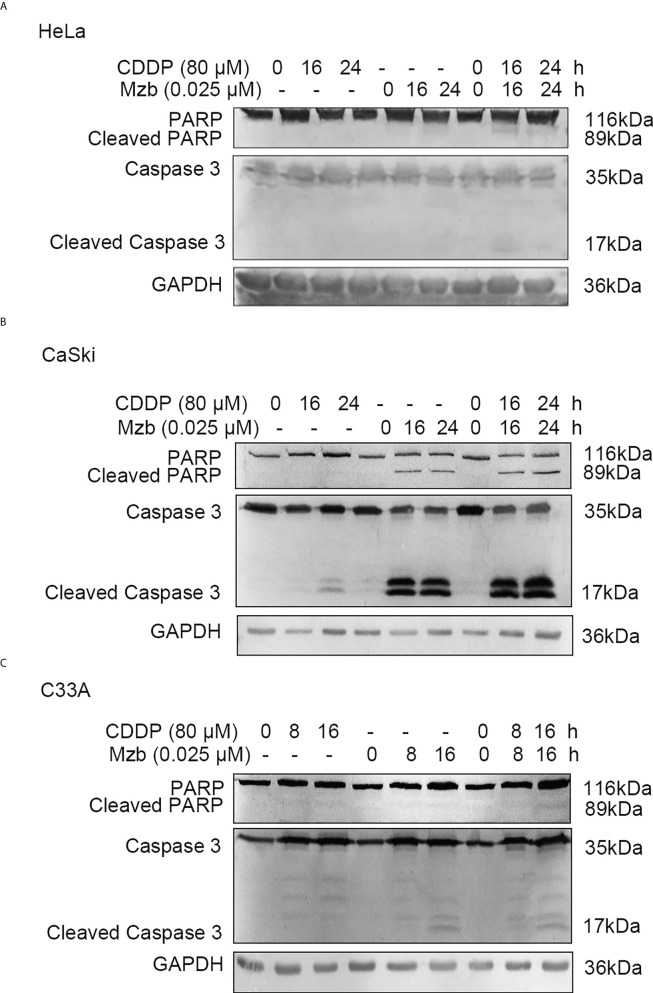
Marizomib strengthens CDDP-induced apoptosis in cervical cancer cells. **(A–C)** The cervical cancer cell lines HeLa and CaSki were treated with CDDP alone (80 μM) marizomib alone (0.025 μM) or the combination of the two drugs for 0, 16 h, or 24 h, and C33A was treated with CDDP alone (80 μM) marizomib alone (0.025 μM) or the combination of the two drugs for 0, 8 h, or 16 h. Then, whole cell lysates were run on SDS−PAGE and immunoblotted with antibodies against PARP and caspase 3 to detect apoptosis. GAPDH was used as the loading control.

To explore whether Mzb could enhance apoptosis of cervical cancer cells induced by CDDP, HeLa and CaSki cells were treated with CDDP alone (80 μM), Mzb alone (0.025 μM) or the combination of the two drugs for 0, 16 h or 24 h. Cleavage of caspase 3 and PARP after Mzb treatment for 24 h was not evident, so C33A cells were treated for 0, 8 h or 16 h. Western blotting analyses indicated that Mzb could promote CDDP-induced PARP and caspase 3 cleavage induced by CDDP in cervical cancer cell lines (**Figures 4A–C**). In order to further verify the combined effect of Mzb and CDDP, three cell lines were treated with 0.025 μM Mzb, 80 μM CDDP or the combination of the two drugs for 16 h or 24 h, respectively, then cell apoptosis rate was detected by flow cytometry. The results revealed that the apoptosis rate was significantly increased after the combined treatment of the two drugs compared with that of the two drugs alone (**Supplementary Figure S1A–C**). These suggest that Mzb could enhances CDDP-induced apoptosis of cervical cancer cells.

### Mzb boosts the cytotoxic effect of CDDP on cervical cancer cells by upregulating Ang-1 expression and downregulating Flt-3L, SCF and Tie-2 expression

**Figure 5:**
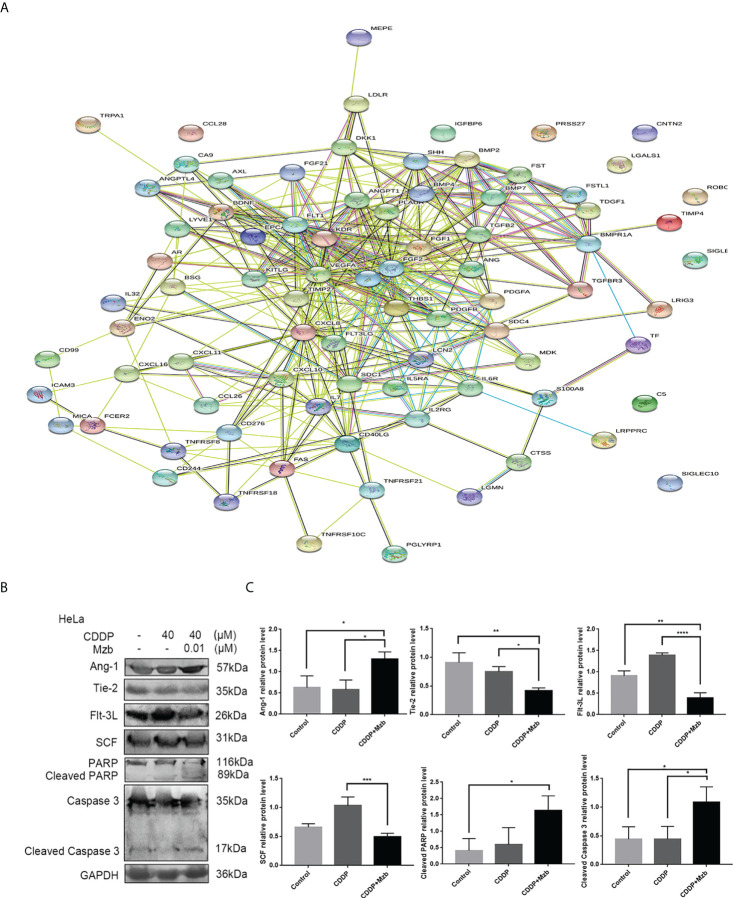
Cytokine array showing that marizomib boosts the cytotoxic effect of CDDP on cervical cancer cells by upregulating Ang-1 expression and downregulating Flt-3L, SCF and Tie-2 expression. The cervical cancer cell line HeLa was treated with CDDP (40 μM) alone or in combination with marizomib (0.01 μM) for 48 h **(A)** The cell supernatant was collected to make a cytokine antibody chip, and the PPI map was obtained. **(B)** The whole cell lysate was then run on SDS−PAGE and western blotted with antibodies against Ang-1, Tie-2, Flt-3L, SCF, PARP, and caspase 3. GAPDH was used as a loading control. **(C)** The gray value was quantified and plotted. *P<0.05, **P ≤ 0.01, ***P ≤ 0.001, ****P ≤ 0.0001, by ANOVA (Dunnett’s multiple comparison post-test).

To investigate the mechanism by which Mzb sensitizes cervical cancer cells to CDDP, a human cytokine array and bioinformatics analysis were performed on HeLa cells, which are less sensitive to CDDP. In the cytokine array, 83 differentially expressed cytokines were identified using one-way ANOVA in the control group, CDDP alone and CDDP combined with Mzb. As shown in **Figure 5A**, the link lines of epidermal growth factor (EGF), fibroblast growth factor 2 (FGF2), kinase insert domain receptor (KDR), Ang-1, Flt-3L and SCF were more abundant, and the numbers of nodes were 36, 36, 27, 15, 11 and 14, respectively. Given that the roles of Ang-1, Flt-3L and SCF in cervical cancer remain unclear, we chose these three factors for further study. Ang-1 and its receptor Tie-2, Flt-3L, and SCF, as well as the apoptosis-related proteins PARP and caspase 3, were detected in HeLa cells (**Figures 5B, C**). The results showed that Mzb combined with CDDP promoted the cleavage of caspase 3 and PARP, upregulated the expression of Ang-1, and downregulated the expression of Flt-3L, SCF and Tie-2 compared with the control and CDDP. In summary, Mzb sensitized apoptosis induced by CDDP and inhibited proliferation by upregulating Ang-1 expression and downregulating Flt-3L, SCF and Tie-2 expression.

### Mzb boosts the inhibitory effect of CDDP on the growth and invasion of HeLa cervical cancer xenografts *in vivo*

**Figure 6:**
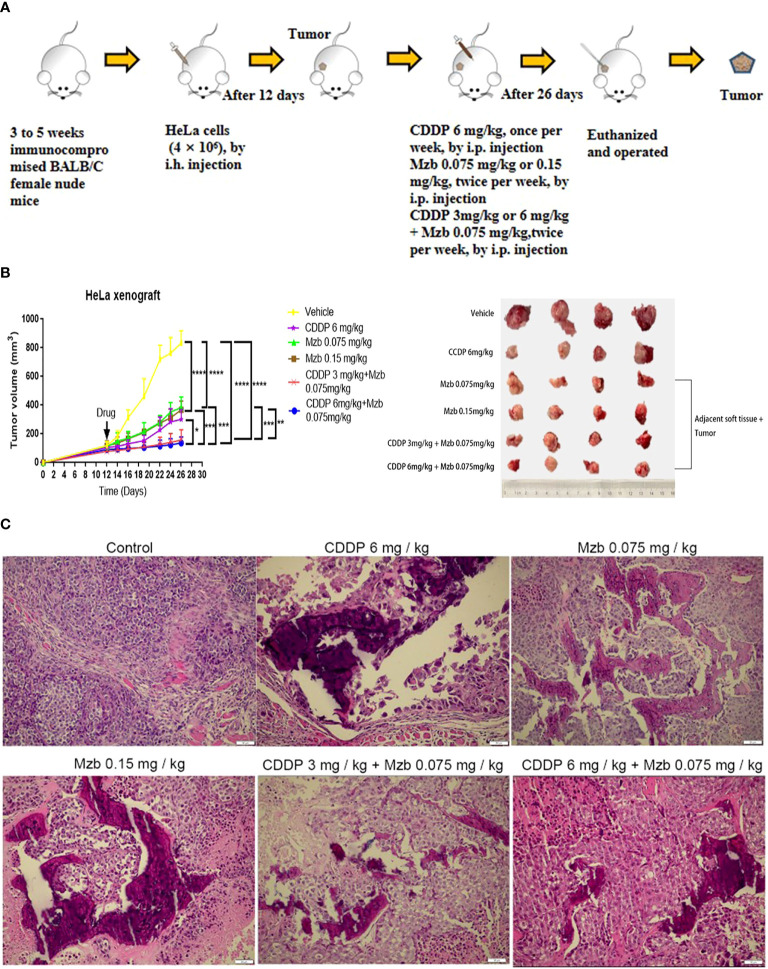
Marizomib boosts the inhibitory effect of CDDP on the growth and invasion of cervical cancer xenografts *in vivo.* **(A)** Schematic of the subcutaneous injection of HeLa cells into the right axilla of female nude mice and drug intervention after tumorigenesis. **(B)** Xenografts were treated with CDDP alone (6 mg/kg), marizomib alone (0.075, 0.15 mg/kg) or a combination of two drugs (CDDP 3 mg/kg and marizomib 0.075 mg/kg, CDDP 6 mg/kg and marizomib 0.075 mg/kg) for 2 weeks, and changes in tumor volume were measured and plotted. *P < 0.05, **P ≤ 0.01, ***P ≤ 0.001, ****P ≤ 0.0001, by ANOVA (Dunnett’s multiple comparison post-test). **(C)** Growth and invasion in the xenografts in each group were observed by H&E staining.

Most proteasome inhibitors sensitize the effect of cytotoxic chemotherapy on cancer cells *in vitro* (28, 29), but their effects in advanced solid tumors are not clear *in vivo*, with high systemic clearance and short half-life, and weaker proteasome inhibitory activity of these drugs in solid tumors (30). Mzb has been shown to irreversibly inhibit all three proteasome active sites in multiple myeloma and in solid tumors (31), thus has increased potency as a multisite antagonist of the proteasome complex compared to other proteasome inhibitors. We further assessed the antitumor activity of Mzb in combination with CDDP on cervical cancer using a nude mice xenograft model. A total of 4×10^6^ HeLa cells were injected subcutaneously into 3 to 5-week-old immunocompromised BALB/c female nude mice (**Figure 6A**). After two weeks of treatment with CDDP, Mzb, or the combination of the two drugs, the mice were sacrificed. Because it was necessary to observe whether Mzb could lower the invasion of cancer cells into the surrounding tissues, the surrounding tissues attached to the tumor bodies were peeled off in the treatment groups. Our results showed that both Mzb and CDDP inhibited tumor growth and invasion as monotherapy compared to the control. At the same time, it is worth noting that Mzb in combination with CDDP significantly inhibited the growth and invasion of xenografts compared with both Mzb and CDDP monotherapy (**Figure 6B**; **Supplementary Figure S2A**). In addition, the mice in the CDDP group lost weight after treatment. Since no significant weight loss was observed in the mice, Mzb alone and the combination with CDDP therapy were well-tolerated (**Supplementary Figure S2B**). Therefore, our data suggest that Mzb combined with CDDP can be used as a better treatment strategy to improve the curative effects in the clinical chemotherapy of cervical cancer in the future.

Necrosis and calcification changes were also observed by H&E staining among the treatment groups, which showed that in the model group, the cell morphology was spindle-shaped, highly atypical, and multinucleated. In addition, the nucleus was deeply stained, the nucleocytoplasmic ratio was increased, mitosis was more common, and tumor cells had a tendency to form glands with a disordered arrangement and an irregular glandular cavity and infiltrated into the surrounding tissue. In the CDDP monotherapy group, the cells lost their original morphology, showed edema, and had a large area of necrosis with calcification but still had a small amount of infringement into the surrounding tissues. In the Mzb low and medium dose and combination groups, the changes in cell morphology were similar to those in the CDDP monotherapy group, whereas necrosis was more pronounced (**Figure 6C**). Therefore, our data suggest that Mzb boosts the inhibitory effect of CDDP on the growth of cervical cancer xenografts and curbs local infiltration.

### Molecular mechanisms of Mzb sensitized CDDP chemotherapy in HeLa cervical cancer xenografts that are relatively insensitive to CDDP

**Figure 7:**
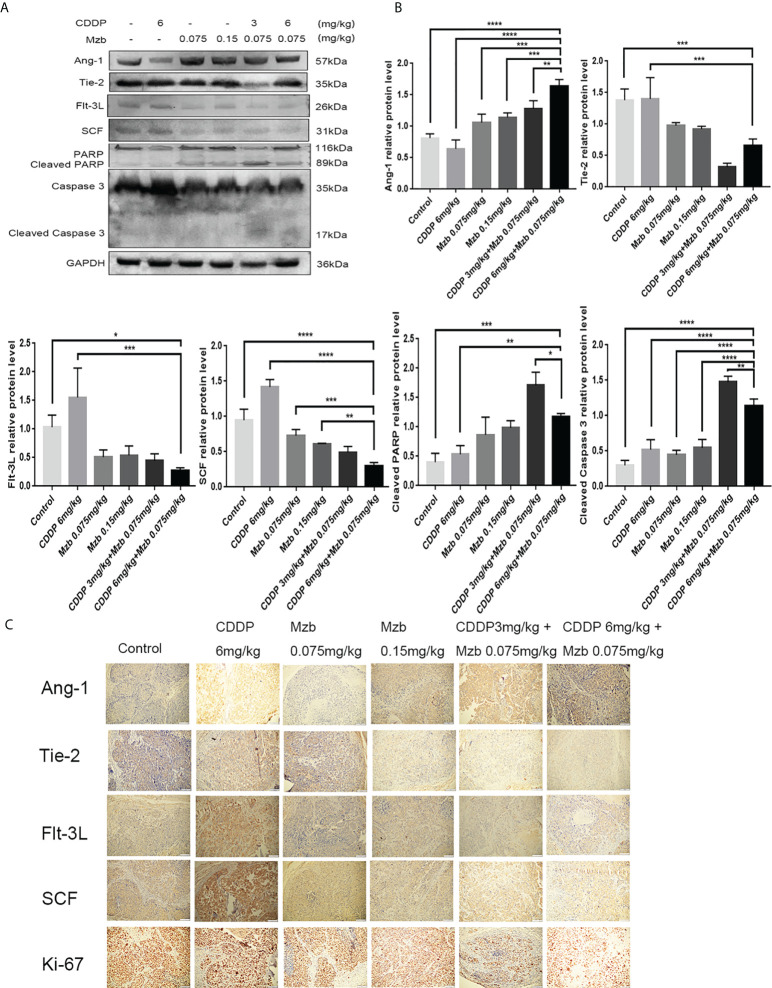
Molecular mechanisms of marizomib sensitized CDDP chemotherapy in HeLa cervical cancer xenografts that are relatively insensitive to CDDP. **(A, B)** The total tissue lysate was then run on SDS−PAGE and western blotted with antibodies against Ang-1, Tie-2, Flt-3L, SCF, PARP, and caspase 3. GAPDH was used as a loading control. *P<0.05, **P ≤ 0.01, ***P ≤ 0.001, ****P ≤ 0.0001, by ANOVA (Dunnett’s multiple comparison post-test). **(C)** Localization and expression of Ang-1, Tie-2, Flt-3L, SCF and Ki-67 in cervical tumors.

We further explored the molecular mechanism by which Mzb sensitized HeLa cervical cancer xenografts that were relatively insensitive to CDDP. The apoptotic-related proteins PARP and caspase 3 and the screened cytokines Ang-1 and its receptor Tie-2, Flt-3L, and SCF were detected in HeLa xenografts. The *in vivo* results were consistent with the *in vitro* results. Compared with the control and CDDP treatment, Mzb alone or in combination with CDDP enhanced the activities of caspase 3 and PARP, upregulated the expression of Ang-1, and downregulated the expression of Flt-3L, SCF and Tie-2. The treatment effect of the combination was more significant (**Figures 7A, B**). Immunohistochemical assays showed that Ang-1, Tie-2, Flt-3L and SCF were located in the cytoplasm and that Ki-67 was localized to the nucleus of cervical cancer cells. The expression of Ang-1 was the highest, and those of Tie-2, Flt-3L, SCF and Ki-67 were the lowest in the combined treatment (**Figure 7C**; **Supplementary Figure S3A–E**). This was consistent with the western blot results. Consequently, the present data suggest that Mzb sensitizes CDDP chemotherapy by upregulating Ang-1 expression and downregulating Flt-3L, SCF, Tie-2 and Ki-67 expression in cervical cancer xenografts.

## Discussion

The proteasome is a large, multicatalytic protein complex that degrades many cellular proteins. Proteasome inhibitors (PIs) are an important new class of drugs for the treatment of multiple myelomas and mantle cell lymphomas, and they are currently in clinical trials for additional types of cancer (32–36). Oprozomib, an oral second-generation PI, increases the sensitivity of cervical cancer cells to apoptosis induced by CDDP (28). Mzb inhibits multiple proteasome catalytic activities and induces a better antitumor response in triple-negative breast cancer cell lines and patient-derived xenografts alone and in combination with standard-of-care chemotherapy (3). In the present preclinical study, we examined the effect of Mzb on inhibiting the proliferation of cervical cancer cells. We found that Mzb has high cytotoxic activity, accompanied by reduced cellular proliferation, attenuated colony forming ability, and increased apoptosis in cervical cancer cell lines representing the major molecular subtypes of cervical cancers, compared with the immortalized cervical epithelial H8 cell line. Although CDDP-based combination chemotherapy is the standard chemotherapy regimen for locally advanced, metastatic and recurrent cervical cancer in the clinic, approximately 50% of cervical cancer patients are commonly resistant to CDDP treatment (37). In light of this, we were interested in investigating the potential function of CDDP treatment in combination with Mzb for cervical cancer *in vitro* and *in vivo.*

Our current results show that Mzb enhances the cytotoxic effect and apoptosis induced by CDDP in cervical cancer cells *in vitro*. Mzb monotherapy showed inhibitory efficacy, to some extent, on cervical cancer growth, and Mzb promoted the suppressive effect of CDDP on the growth of subcutaneous cervical cancer xenografts in nude mice *in vivo*. These results suggest that Mzb monotherapy can inhibit cervical cancer growth, and that treatment with Mzb and CDDP shows strong anticancer activity *in vivo*. Mzb is currently being tested in several hematological malignancies and solid tumors both as a monotherapy and in combination with standard-of-care chemotherapy. Raninga PV et al. reported that Mzb induces a better antitumor response in triple-negative breast cancer cell lines and patient-derived xenografts alone and in combination with standard-of-care chemotherapy (3). Boccellato C et al. reported a high efficacy of combination treatments with a TRAIL receptor agonist and Mzb in relevant glioblastoma cell models (4). The results of our assay are consistent with the above. Several studies are testing Mzb for glioblastomas on the basis of the ability of this drug to cross the blood–brain barrier. To the best of our knowledge, except for multiple myeloma, glioblastoma and other hematological malignancies, studies of Mzb for solid tumors in clinical trials have not been reported.

While the primary action mechanism of proteasome inhibitors is the inhibition of catalytically active subunits, the up-stream molecular events that lead to selective cell death and sensitize the effect of chemotherapeutic drugs on cancer are not clear. In the present study, we performed a cytokine antibody chip test in order to explore the molecular mechanism of the therapeutic effect of Mzb sensitizing CDDP on cervical cancer. Three differential proteins, Ang-1, Flt-3L, and SCF, were identified based on KEGG and protein−protein interaction network (PPI) analyses in STRING data. *In vitro* and *in vivo*, the expression of the pro-apoptotic molecule Ang-1 was significantly increased in the combination group, and the expression of the pro-proliferation molecules Flt-3L and SCF was significantly reduced in the combination group. In addition, the expression of Tie-2, an Ang-1 receptor, was decreased in the combination group. We provide evidence that Mzb promotes apoptosis and inhibits proliferation by promoting Ang-1 expression and inhibiting the expression of Flt-3L, SCF and Tie-2 to sensitize cervical cancer to CDDP treatment *in vitro* and *in vivo*. As shown, the CIs for most combinations of Mzb and CDDP were far lower than 1.0, indicating synergistic effects of these two drugs on cervical cancer cells. The combination of the lower doses of Mzb and CDDP significantly and synergistically induced increasingly cytotoxic effects and apoptosis in cervical cancer cells and inhibited the xenograft growth of cervical cancer in nude mice in our assays.

Dysregulation of Angs, ligands of the tyrosine kinase receptor Tie-2, has been associated with a number of diseases, including cancer (38, 39). Ang-1 was found to be essential for the maturation and “sealing” of newly formed blood vessels (40). Due to insufficient levels of Ang-1 in cancer patients, tumor angiogenesis generates unsealed “leaky” blood vessels (41). Multiple studies have demonstrated that Ang-1 actually inhibits tumor growth. A high serum Ang-1 level was associated with a longer progression-free survival and a longer overall survival in cervical cancer patients (38). Ang-1 can increase the infiltration of T cells and improve the prognosis of endometrial carcinoma patients (41). In our *in vitro* and *in vivo* experiments, CDDP blocked the apoptosis of HeLa cells by inhibiting the expression of Ang-1, which is one of the possible molecular mechanisms of CDDP resistance in HeLa cells. Mzb promoted the expression of Ang-1, thus attenuating the CDDP resistance of HeLa cells. Tie-2 was overexpressed in both HeLa cells and xenografts in our experiments and was significantly weakened by treatment with Mzb combined with CDDP. This suggests that combined treatment with Mzb and CDDP promoted Ang-1 expression and lowered Tie-2 expression, thus inhibiting angiogenesis and contributing to vascular maturation, resulting in an obvious anticancer effect.

**Figure 8:**
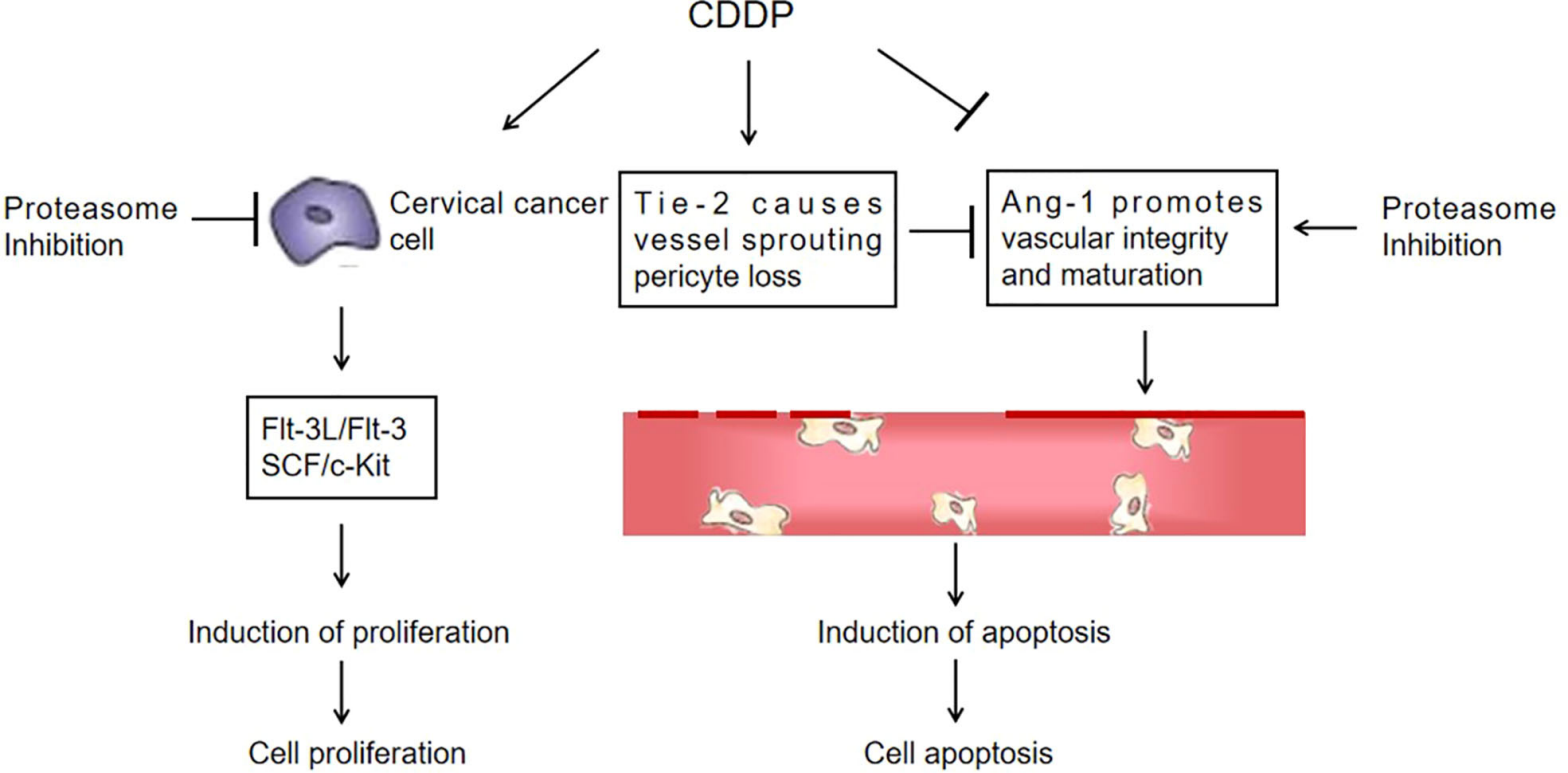
A proposed schematic of the cytotoxic effect of marizomib on cervical cancer. The illustration depicts the proposed signaling events from proteasome inhibition, leading to the upregulation of Ang-1 expression and the downregulation of Flt-3L, SCF and Tie-2 expression in cervical cancer xenografts, promoting vascular normalization and ultimately cell death.

Flt-3L is a type I transmembrane protein that can be released as a soluble homodimeric protein (42). Elevated Flt-3L levels have been reported in several cancers, such as in the serum of colorectal cancer (43) and prostate cancer patients (44). In addition, Flt-3L levels are abnormally increased in the majority of leukemias (45). The basal level of Flt-3L was significantly lower after bevacizumab treatment with paclitaxel in patients with increased Flt-3L in advanced ovarian cancer (46). Flt-3L plays a positive role in promoting cancer progression. Our data supported that Flt-3L expression was increased in cervical cancer cells and tissues and markedly reduced in the combination therapy of CDDP with Mzb. Consequently, CDDP combined with Mzb may be beneficial in lowering this cytokine level. The mitogenic cytokine SCF is a proinflammatory glycoprotein that upon homodimerization binds to the c-Kit receptor (Kit gene, CD117) and exists both as a membrane-bound and soluble form (47), and c-Kit is a well-known oncogene. Activation of c-Kit by SCF binding promotes survival, proliferation, stemness, immune evasion and drug resistance of malignant cells (48, 49). SCF is considered an adverse prognostic marker in cancer. In our study we also found increased SCF expression in cervical cancer cells and xenografts and decreased SCF expression in the combination of CDDP with Mzb. Our data support the model described in **Figure 8**. This finding demonstrates that although cervical cancer cells showed higher sensitivity to Mzb, cervical cancer cells were further sensitized by the combination of Mzb with CDDP treatment. Mzb may sensitize cervical cancer cells to CDDP chemotherapy and thus lessen CDDP resistance and toxicity. This lays a foundation for the further development of inhibitors for new targets for clinical synergistic drugs.

In conclusion, based on the data *in vitro* and *in vivo* using a panel of cervical cancer cell lines and nude mice, we provided compelling evidence that Mzb monotherapy can suppress proliferation and induce apoptosis and enhance the cytotoxic effect and apoptosis induced by CDDP in combination by strengthening Ang-1, inhibiting Tie-2 expression in the Ang-1/Tie-2 signaling pathway, and inhibiting the Flt-3/Flt-3L and SCF/c-Kit proliferative signaling pathways. This study suggests that the combination of CDDP with Mzb produces synergistic anticancer activity and that Mzb may be a potential effective drug in combination therapy for cervical cancer patients.

## Data availability statement

The raw data supporting the conclusions of this article will be made available by the authors, without undue reservation.

## Ethics statement

The animal study was reviewed and approved by The Animal Laboratory Center of Xinjiang Medical University.

## Author contributions

YS developed the study concept and design. ZZ, SZ, BL, QW and XN performed the experiments and collected the data. ZZ, YS and SZ analyzed the data. ZZ and YS drafted the manuscript. All authors contributed to the article and approved the submitted version.

## Funding

This work was supported by the Natural Science Foundation Project of Xinjiang Autonomous Region, China (2019D01C221, 2021D01A47, to YH-S).

## Conflict of interest

The authors declare that the research was conducted in the absence of any commercial or financial relationships that could be construed as a potential conflict of interest.

## Publisher’s note

All claims expressed in this article are solely those of the authors and do not necessarily represent those of their affiliated organizations, or those of the publisher, the editors and the reviewers. Any product that may be evaluated in this article, or claim that may be made by its manufacturer, is not guaranteed or endorsed by the publisher.
