# Supplementary material for: Combined treatment of marizomib and cisplatin modulates cervical cancer growth and invasion and enhances antitumor potential in vitro and in vivo
Source: Front Oncol. 2022 Aug 30;12:974573. doi: 10.3389/fonc.2022.974573 (PMC9468930; doi:10.3389/fonc.2022.974573)
Supplement: Supplementary file 1 [file DataSheet_1.pdf]

Supplementary Materials

Supplementary Tables and Figures

Table S1 The combined cytotoxic effect of marizomib and CDDP for the combination indexes (CIs) values and their inhibition rates in HeLa cell line

Dose Mzb (μM)	Dose CDDP (μM)	Effect	CI
0.01	0.3	0.001	1.48
0.01	0.6	0.026	0.81
0.01	5	0.053	0.56
0.01	10	0.167	0.29
0.01	20	0.261	0.22
0.01	40	0.445	0.14

Table S2 The combined cytotoxic effect of marizomib and CDDP for the combination indexes (CIs) values and their inhibition rates in CaSki cell line

Dose Mzb (μM)	Dose CDDP (μM)	Effect	CI
0.01	0.3	0.814	0.25
0.01	0.6	0.839	0.21
0.01	5	0.884	0.13
0.01	10	0.897	0.12
0.01	20	0.884	0.13
0.01	40	0.87	0.16

Table S3 The combined cytotoxic effect of marizomib and CDDP for the combination indexes (CIs) values and their inhibition rates in C33A cell line

Dose Mzb (μM)	Dose CDDP (μM)	Effect	CI
0.01	0.3	0.39	1.11
0.01	0.6	0.416	0.9
0.01	5	0.489	0.51
0.01	10	0.535	0.37
0.01	20	0.554	0.33
0.01	40	0.555	0.37

Table S1-S3: Marizomib enhances the cytotoxic effect of CDDP on cervical

cancer cells. The data were analyzed in CCK-8 assays for the combined cytotoxic effect of marizomib and CDDP for calculating the combination indexes (CIs) values and their inhibition rates were listed.

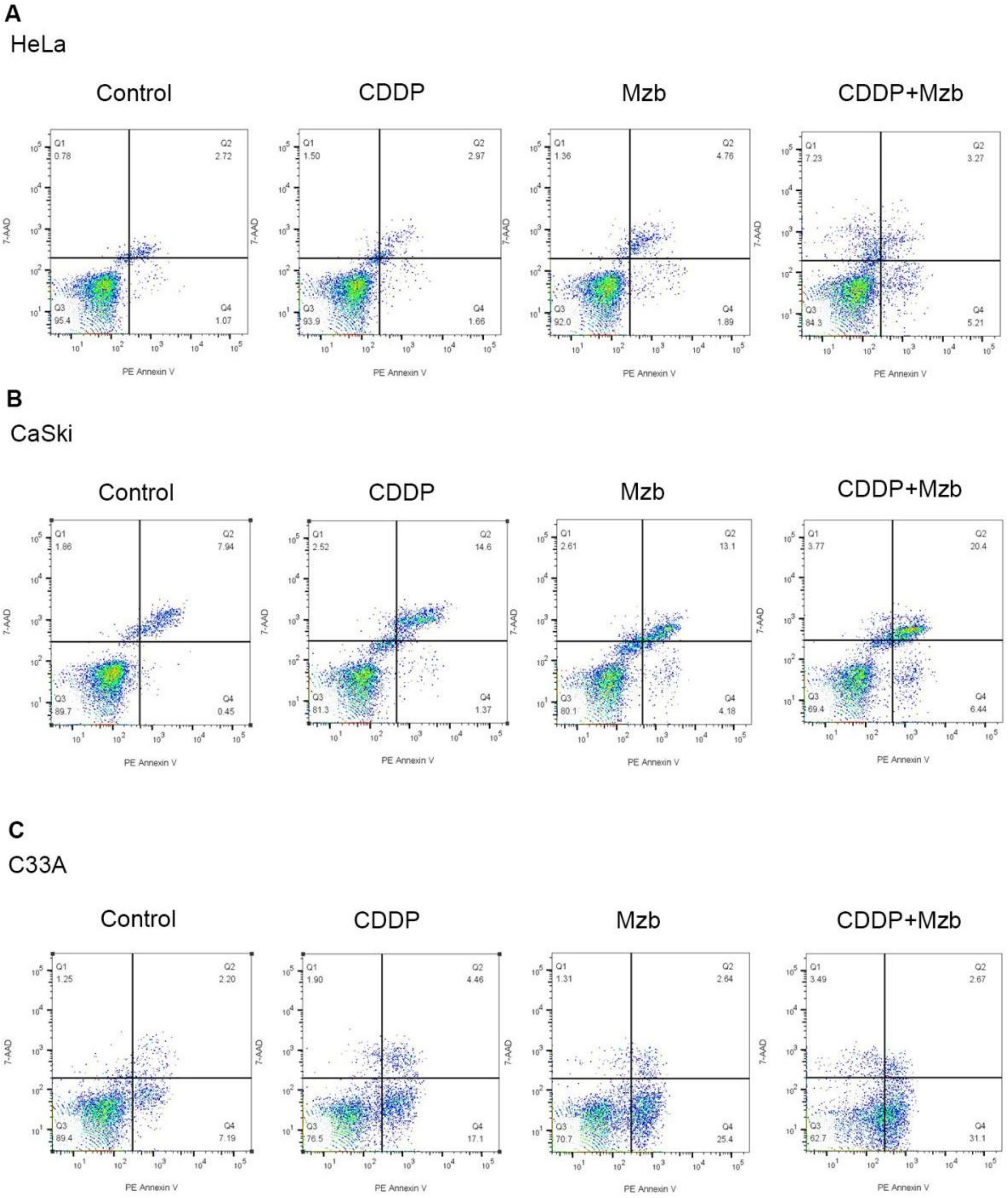

Figure S1: Marizomib enhances CDDP-induced apoptosis of cervical cancer cells. A-C. The results of the flow cytometry of HeLa, CaSki and C33A cells treated with CDDP, marizomib or the combination of the two drugs were shown. The cells were incubated in 6-well plates at 1×10^6 cells per well with CDDP (80 μM), marizomib (0.025 μM) or the combination of the two drugs for 16 h or 24 h, then analyzed by flow cytometry for the percentage of apoptotic cells after incubating with PE and 7-AAD for 15 min at RT.

A

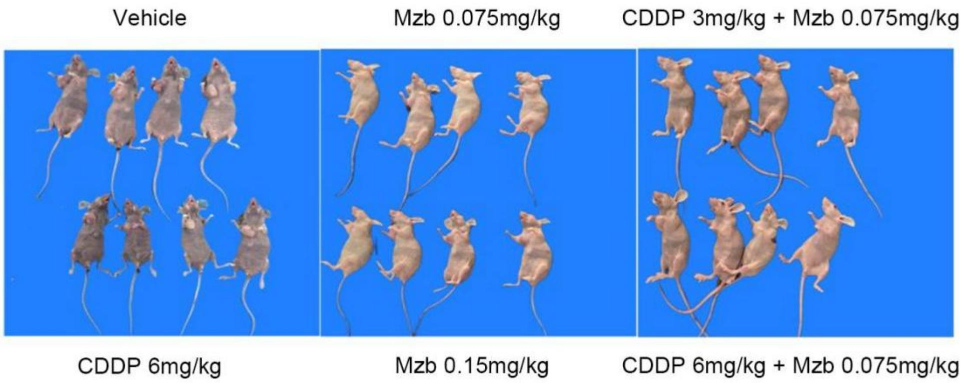

B

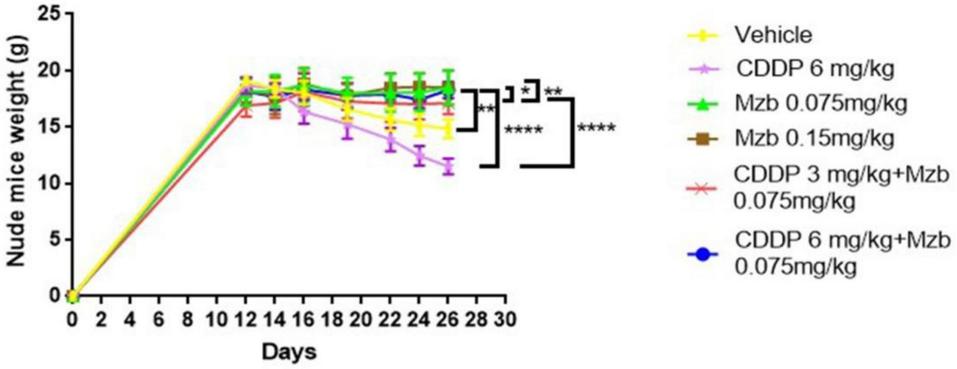

Figure S2: Marizomib boosts the inhibitory effect of CDDP on the growth and invasion of HeLa cervical cancer xenograft *in vivo.* A. Xenografts were formed after HeLa cells were injected into mice subcutaneous for 2 weeks. Then, the mice were randomly divided into 6 groups (4/group) and then treated with PBS (once per week, by i.p. injection), CDDP (6 mg/kg, once per week, by i.p. injection), Mzb (0.075 mg or 0.15 mg/kg, twice per week, by i.p. injection), or a combination of two drugs (CDDP 3 mg/kg, Mzb 0.075 mg/kg; CDDP 6 mg/kg, Mzb 0.075 mg/kg; twice per week, by i.p. injection) for 2 weeks. B. The mice in CDDP group lost their weight after treatment compared with before treatment. Since no significant weight loss was observed in the mice among other ones, marizomib alone and the combination with CDDP therapy were well tolerable. *$P<0.05$, **$P\leq0.01$, ***$P\leq0.001$, ****$P\leq0.0001$, by ANOVA (Dunnett's multiple comparison post-test).

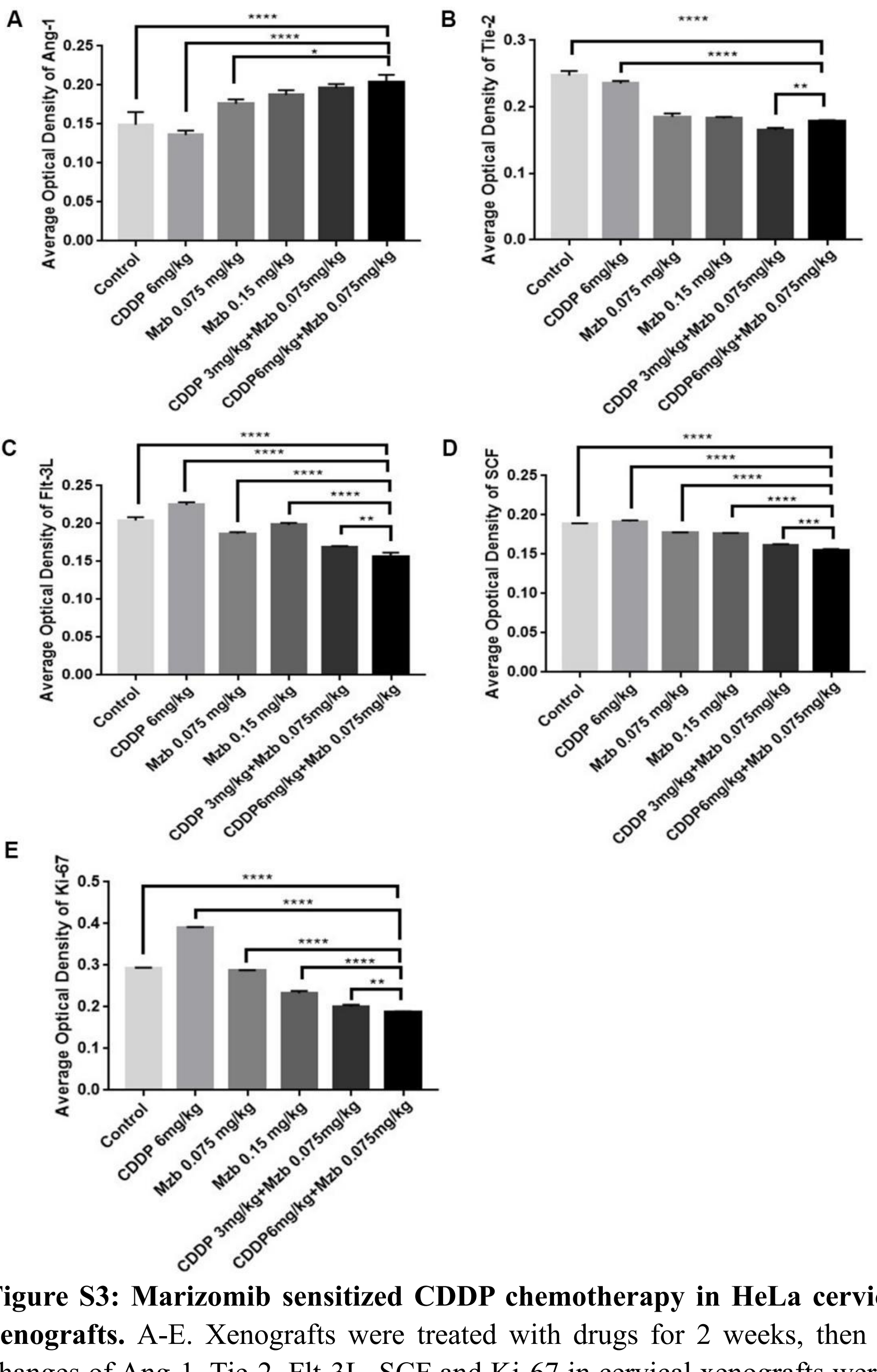

Figure S3: Marizomib sensitized CDDP chemotherapy in HeLa cervical cancer xenografts. A-E. Xenografts were treated with drugs for 2 weeks, then expression changes of Ang-1, Tie-2, Flt-3L, SCF and Ki-67 in cervical xenografts were observed by immunohistochemistry and analyzed by Image J. *P<0.05, **P ≤ 0.01, ***P ≤ 0.001, ****P≤0.0001, by ANOVA (Dunnett's multiple comparison post-test).
